# Boning up: amino-bisphophonates as immunostimulants and endosomal disruptors of dendritic cell in SARS-CoV-2 infection

**DOI:** 10.1186/s12967-020-02433-6

**Published:** 2020-06-29

**Authors:** Adam Brufsky, Juan Luis Gomez Marti, Azadeh Nasrazadani, Michael T. Lotze

**Affiliations:** 1 UPMC Hillman Cancer Center, Magee Women’s Hospital, University of Pittsburgh, School of Medicine, Suite 4628, 300 Halket Street, Pittsburgh, PA 15213 USA; 2 University of Pittsburgh School of Medicine, Pittsburgh, PA 15213 USA; 3 UPMC Hillman Cancer Center, Pittsburgh, PA 15213 USA; 4 Department of Surgery, UPMC Hillman Cancer Center, Rm G.27A, 5117 Centre Avenue, Pittsburgh, PA 15213 USA

**Keywords:** SARS Coronavirus, Immune response, Glycoproteins, Bisphosphonates

## Abstract

Amino-bisphosphonates such as zoledronic acid (ZA) can possibly ameliorate or prevent severe COVID-19 disease by at least three distinct mechanisms: (1) as immunostimulants which could boost γδ T cell expansion, important in the acute response in the lung; (2) as DC modulators, limiting their ability to only partially activate T cells; and (3) as prenylation inhibitors of small GTPases in the endosomal pathway of the DC to prevent expulsion of lysosomes containing SARS-CoV-2 virions. Use of ZA or other amino-bisphosphonates as modulators of COVID-19 disease should be considered.

## Zoledronic acid as an immunostimulant of γδ T cells

Zoledronic acid (ZA) is a nitrogen containing aminobisphosphonate with wide use in breast cancer in patients taking aromatase inhibitors to prevent osteoporosis [1]. ZA was first noted in 2009 to reduce the incidence of bone metastases in post-menopausal women [2]. Large clinical trials [3] and a meta-analysis [4] confirmed the benefit of ZA in early stage post-menopausal breast cancer, with highly significant reductions in distant recurrence, bone recurrence, and breast cancer mortality.

**Fig. 1:**
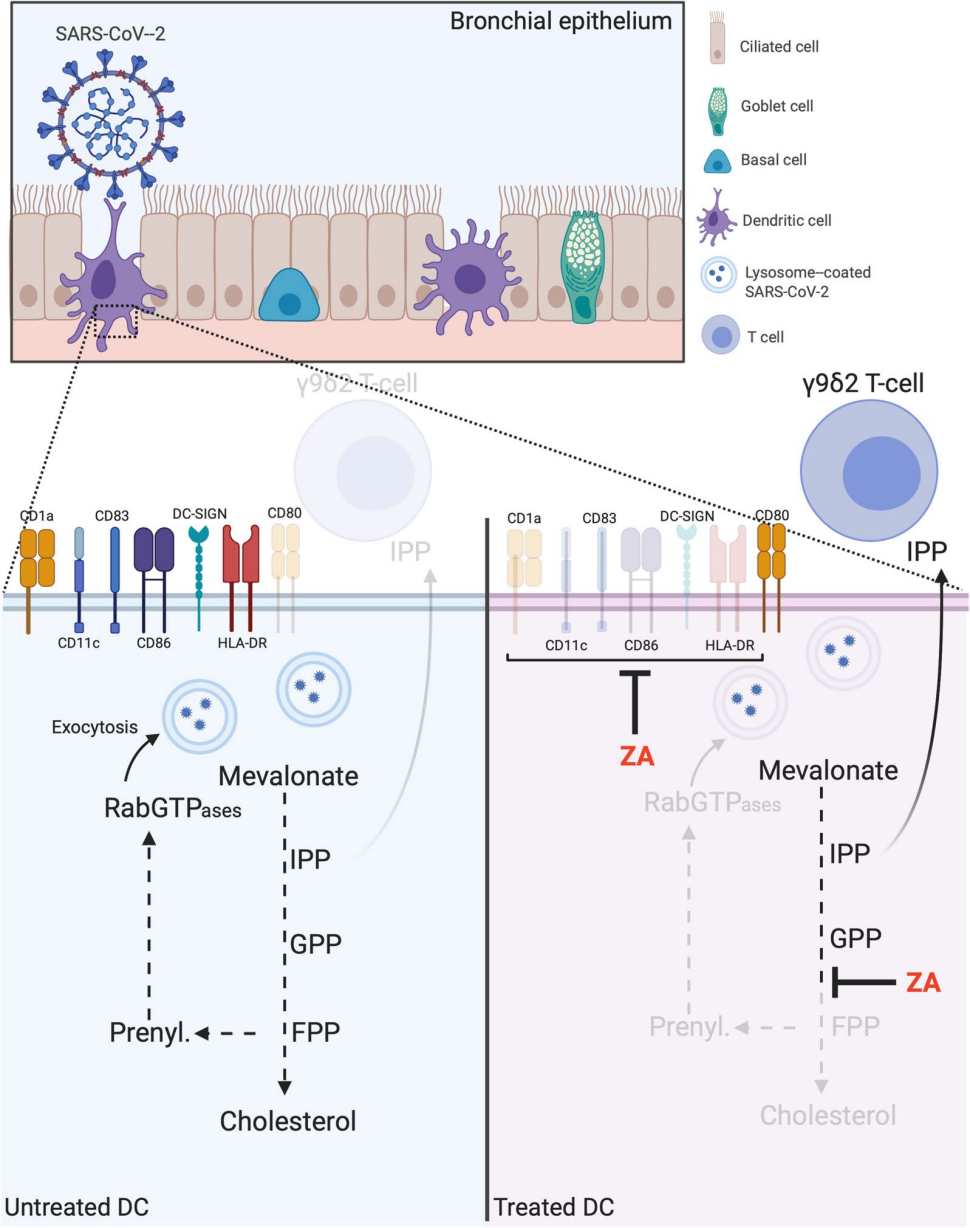
Zoledronic acid (ZA) acts as immunostimulant and endosomal disruptor of dendritic cell in SARS-CoV-2 infection. Inhaled SARS-CoV-2 particles are internalized by the DC (top). In COVID-19 disease, there is depletion of γδ T cells (bottom-left). In addition, virion release depends on prenylation signaling derived from the mevalonate pathway. On the other hand, ZA (bottom-right) inhibits the conversion of geranyl pyrophosphate (GPP) to farnesyl pyrophosphate (FPP), increasing the concentrations of isopentenyl pyrophosphate (IPP). Release of IPP induces γ9δ2 T-cell expansion by phosphoantigen recognition, mediated by butyrophilin-presentation. Downstream inhibition of prenylation reduces the activity of GTPases, decreasing the release of SARS-CoV-2. ZA also affects differentiation of the DC with downregulation of the expression of CD1a, CD11c, CD83, CD86, DC-SIGN, and HLA-DR and enhancement of the expression of CD80. Figure was created using BioRender https://biorender.com/

ZA inhibits the mevalonate pathway (Fig. 1) through inhibition of farnesyl diphosphate synthetase, leading to upstream accumulation of phosphoantigen isopentyl diphosphate (IPP), stimulating γ9δ2 T cell expansion [5]. Some γδ T cells have direct cytotoxicity against breast cancer cells in vitro [6]. In early stage breast cancer, treatment with a single dose of ZA results in long lasting activation of effector subsets of γ9δ2 T lymphocytes [7]. ZA can also increase natural killer (NK) cells through a DC mediated mechanism modulated by γ9δ2 T cells [8]. Approximately 50% of patients taking ZA and other aminobisphosphonates experience an acute phase reaction [9], which is correlated with γδ T cell subset expansion [10].

T cells that express gamma/delta (γδ) T cell receptors are important in the immune response to viruses [11] as well as cancer [12]. In the setting of HIV infection, the circulating γ9δ2 T cells, most responsive to butyrophilin presented mevalonate pathway molecules, are eradicated for years even with successful control of viral infection [13]. Three months after recovery from SARS, patients exhibited selective expansion of γδ T cell populations, but not alpha/beta (αβ) T cell populations [14]. γδ T cell expansion is associated with higher anti-SARS-CoV IgG titers [14]. When non-MHC restricted γδ T cell are stimulated with SARS-CoV in vitro, they kill SARS-Co-V infected THP-1 myeloid cells in culture with high efficiency. This suggests a protective effect of γδ T cells in SARS-CoV infection. Agents which expand γδ T cell populations within the lung [14] could protect individuals from COVID-19 and/or ameliorate symptoms.

Pamidronate reduces disease severity and mortality from human H1N1 influenza virus and avian H5N1 influenza virus in an immunodeficient mouse–human immune chimera [15] through expansion of γδ T cell subsets. Inhaled intranasal liposomal clodronate can reduce inhibitory alveolar macrophages and increase respiratory DC migration and activation in a mouse model of SARS-CoV [16]. This can reduce viral titers and protect the animals from lethal infection.

Concerns regarding the clinical consequences and safety of γδ T cell expansion are valid. In preclinical models, Oberg et al. [17] demonstrate the successful recruitment of γδ T cells to sites of tumor in a model of pancreatic adenocarcinoma by which tumor growth is retarded in immunocompromised mice. These mice may not be able to manifest the full consequences of immune activation. In human studies, no major toxicities were appreciated in a phase I trial of metastatic hormone-refractory prostate cancer patients in which ZA was utilized to activate Vγ9 Vδ2 T cells with or without IL-2 **[**18]. ZA expanded γδ T cells were additionally noted to be safe in patients with NSCLC [19] following adoptive transfer.

The anti-viral effects of γδ T cells, mediated by release of infected-cell specific IFN-γ [20] promotes an anti-SARS-CoV (2003 outbreak) effect with expansion of γδ-cells [14]. T cells, including γδ subpopulations, are depleted in patients with severe COVID-19 illness [21]. Indeed stimulation of γ9δ2 T cells early in the disease would be safe but would require careful analysis of the cytokine profiles after γδ T cell-expansion. Recent findings in malaria models suggest that M-CSF production from γδ T cells is important for long-lasting control of the disease [22]. Such an M-CSF producing cell may be important in regulating and controlling the SARS-CoV2 virus.

## The respiratory DC as central to SARS-CoV-2 pathogenesis

In the SARS-CoV-2 infection, there is initial lymphopenia. The lymphocyte count is predictive of disease severity and mortality [23]. Lymphocyte counts recover with viral clearance and disease resolution, with adaptive immune cells (CD3+ T cells) being especially important [24]. Such immune deficiency can in part be explained by viral infection and T cell interaction with the respiratory DC.

Early and central infection of tissue resident dendritic cells (DC) by the SARS-CoV-2 coronavirus explain some of the immunopathology of the COVID-19 pandemic. DC are richly abundant in the lung and responsive to viral infection [25]. In COVID-19, T cell receptor (TCR) repertoires are dramatically reduced during the early onset of severe SARS-CoV-2 infection but recover during the convalescent stage [26]. Such reduction of T cells suggest acute wholesale apoptotic death with engagement of the TCR in the absence of costimulatory molecules, normally provided by DC [27].

## Zoledronic acid as a dendritic cell modulator

ZA treatment can affect DC differentiation and maturation, decreasing expression of CD1a, CD11c, CD83, CD86, DC-SIGN, HLA-DR and enhancing expression of CD80 [28]. ZA can augment the allostimulatory activity of DCs [28]. ZA can also activate human NK cells in a DC dependent but γδ T cell independent manner [8]. ZA could therefore act on the DC to further stimulate the initial immune response to pathogens such as SARS-CoV-2.

The interplay between γδ T cells and dendritic cells is indeed complex [29]. Depending on the clinical setting and the pathogen involved, such interaction can generate and inhibitory or stimulatory immune response.

In regard to the connection between DC and γ9δ2 cells, it is important to point out that Calmette-Guerin recognition by dendritic cells promotes the expansion of γ9δ2 cells, which occurs via isopentyl diphosphate (IPP) release [30]. In this regard, ZA also prompts IPP release from dendritic cells. This potent inhibitor of farnesyl diphosphate synthase is known to induce the release of IPP from dendritic cells when these are treated with ZA [31]. In fact, 1-Hydroxy-2-methyl-2-buten-4-yl 4-diphosphate (HDMAPP) is the most potent phosphoantigen that stimulates γ9δ2 cells. This appears to a critical mechanism of chemical synapse between DCs and γδ cells.

## Zoledronic acid as a disruptor of the endosome in SARS-CoV-2 infection

While virus replication of SARS-Co-V within infected human monocytes is limited, the virions are observed by electron microscopy to accumulate in phagolysosomes of the endosomal system [32]. Some of the potential immune dysfunction of DCs in SARS-CoV-2 infection can therefore be traced to the endosome. The endosomal pathway is critically important in DC antigen function, processing, and cross presentation of antigens [33]. Proper ion and pH homeostasis in the endosomal compartment appears to be required for glycosylation of proteins, membrane trafficking, and protein sorting [34].

It is worth noting that preclinical screening of anti-SARS-CoV-2 drugs identified three agents, cepharanthine (CEP), selamectin, and mefloquine hydrochloride [35] that could disrupt endosomal pH through alterations in ion balance. Endosomal homeostasis is likely important in SARS-CoV-2 pathogenesis. The active endosomal environment central to DC biology could be exploited by SARS-CoV-2. Agents that alter endosomal pH such as hydroxychloroquine (HCQ) could be protective in SARS-CoV-2 infected DCs in maintaining the immune response as well as the lymphocyte count, as was observed in a recently reported randomized, parallel, open label, multicenter clinical trial of hydroxychloroquine (HCQ) and usual care versus usual care alone for the treatment of COVID infection [36].

The likely involvement of the endosome in SARS-CoV-2 infection is suggested by a possible attenuation mutation in the ORF 3a protein. Viral evolutionary theory suggests that one option for a viral strain introduced to a novel host is to maintain fitness through reduction in virulence [37]. The ORF 3a protein of SARS-CoV is comprised of 247 amino acids with three transmembrane domains [38]. It is a putative ion channel that is present both in the endosomal compartment and within the cell membrane [38], modulating release of virus [38]. Deletion mutants of ORF 3a demonstrate its importance in SARS-CoV virulence in mice [39]. Emodin, an ion channel inhibitor, blocks SARS-CoV pathogenesis in culture and inhibits viral release from the cell [40]. A stable Q57H non-synonymous substitution in the ORF 3a protein appears to have arisen in a subclade of D614G mutant SARS-CoV-2 [41]. This substitution is near the N-terminus of transmembrane region I of ORF 3a [39] and is predicted to be deleterious [42]. A mutation in one of the ion channels of the SARS-CoV-2 ORF 3a protein could possibly inhibit release of the virus by phagolysosomes” and, by this mechanism, reduce virulence.

The importance of the endosome in SARS-CoV-2 pathogenesis is also underscored by a recent report [43], where 26 of the 29 SARS-CoV-2 proteins in human cells were tagged and identified as being physically associated using affinity-purification mass spectrometry (AP-MS). This assay identified 332 high-confidence SARS-CoV-2-human protein–protein interactions (PPIs), and approximately 40% of the SARS-CoV-2 interaction proteins were associated with the endomembrane compartments or vesicle trafficking pathways.

ZA could plausibly attack the endosomal trafficking central to SARS-CoV-2 infection. ZA inhibits the prenylation of small guanine-nucleotide-binding regulatory proteins (G-proteins) such as Rab family members through inhibition of geranylgeranyl transferases [44, 45]. Rab GTPase family members are involved in endosomal trafficking, including compartmentalization into early, recycling, late, and lysosomal routes [46]. Osteoclasts require endosomal trafficking, lysosomal sorting, and exocytosis of lysosomes for the secretion of the hyaluronidase HYAL1, which degrades bone collagen and can cause osteoporosis [47]. ZA administration can disrupt such trafficking in osteoclasts through inhibition of G-protein geranylation [48]. Since osteoclasts and DCs share a common precursor as well as many functions [49, 50] it is plausible that ZA would inhibit endosomal pathway and exocytosis in the DC as well, and possibly prevent trafficking and exocytosis of lysosomes in the SARS-CoV-2 virion-infected DC.

We propose consideration of ZA as therapy for COVID-19 if given early in the disease course, preferably following exposure and before symptoms occur. While ZA is currently given by intravenous infusion, oral preparations are in development [51]. Oral aminobisphosphates such as ibandronate also expand γδ T cell subsets [52].

## Conclusion

Infection of the DC by SARS-CoV-2 could explain the exuberant distal immunopathology observed in COVID-19 [25]. The immune-depleted environment as a result of early infection is a possible setting for therapeutic intervention. Amino-bisphosphonates may be capable of making DCs or DC precursors less susceptible to further SARS-CoV-2 infection. Immunostimulating γ9δ2 T cell expansion, DC membrane receptor modulation with NK activation, and prenylation inhibition of small GTPases with consequent inhibition of endolysosomal pathways used in the viral lifecycle are all plausible mechanisms of actions of ZA in this context. In the absence of clinical data, it is unclear if ZA alone would sufficiently achieve these intended goals. Hence, these data form the foundation for clinical trials.

In conclusion, possible amelioration of the immune host status through amino-bisphosphonate use should be considered for COVID-19 disease.

## Data Availability

Not applicable.
